# Echoes of the Month

**Published:** 1924-05

**Authors:** 


### ECHOES OF THE MONTH.

### CHILDREN ADDICTED TO COCAINE.

There are reported to be in Moscow alone 50,000 homeless children, and about 40 per cent. of them are found to be cocaine addicts. Their ages range from eight to thirteen. In one instance twenty children were taken at random from one of the leading juvenile labour communes, and every one was discovered to be suffering from the drug. Doctor Bocharov, one of Moscow's noted pathologists, declares that he found that many children who spent four to ten pounds weekly on cocaine obtained the money almost entirely by stealing. A large number of them live in the outskirts of the city, where they have formed themselves into labour communes. Some of these places have become veritable cocaine dens, where frequently very small children lie entranced for days under the influence of the drug.

### A CORONER'S EXPERIMENT.

Dr. Ambrose, the Coroner for South-West Essex, is engaging a room or hall between Leyton and Walthamstow where people can obtain his advice and tell him of their troubles. In the course of his duties Dr. Ambrose has to investigate numerous tragedies which are the outcome of people suffering from "nerves" and worry. He firmly believes that if people on the border of nervous prostration through worry can be seen by someone to whom they can unburden themselves, it would be much comfort to them, and perhaps be the means of preventing them taking a drastic and fatal step. In the last eighteen months of the war Dr. A. Ambrose worked in a hospital dealing with cases of shell-shock, at which he gained much experience. Since then he has come into personal contact with similar cases, and in several he had talked to men and at least put the idea of suicide out of their heads

### FIVE GREAT SOURCES OF DISEASE.

Speaking at Sheffield on "Some Moral Aspects of Public Health," Sir Arthur Newsholme said that the improvement in public health which had taken place in the last fifty years was the more remarkable when it was remembered that a great many more people lived in town areas at present than in the country. How was it then that these vast improvements had been brought about? Firstly, by the discovery of the vectors of infection, such as yellow fever, malaria and typhoid fever; and secondly, the immunity against infection by vaccination as in the case of small-pox. Then, of course, we must not forget the improvements made in our water and milk supplies, the admirable treatment afforded in our hospitals, and also the wonderful work performed by our medical officers of health all over the country. But even though such great strides had been made, there were five great sources of disease that we had not yet conquered—catarrhal infections, cancer, tuberculosis, alcoholism and venereal disease, and the last four of those dread scourges were the most prolific causes of mortality at present in this country that we had not overcome, but which we should strive by all means in our power and the exercise of our intelligence to kill.

### MEDICAL MEN AND PRIVATE HOSPITAL PATIENTS.

Writing in the *Empire Review* Lord Hambleden, Chairman of King's College Hospital, comments on "the close co-operation between the medical board and the committee of management which has enabled a further development to be made in an experimental way, which appears to be quite exceptional in the reception of private patients." Many patients know of no special surgeon or physician whose services they desire and "are content with the assurance that they will be in the care of a member of the hospital staff." The financial arrangements for their reception are left entirely to the chief administrator, the House Governor, who is a layman, and acts as an intermediary between the patient and the medical man merely so far as regards the payment and collection of the fees. It has been possible through that means to relieve the patient or his friends of the chief anxiety in the time of illness, namely as to the amount of expenditure to be incurred. If the patient comes into hospital for a straightforward operation it is possible to tell him that the total amount of his liability will be for example, forty guineas, and if his circumstances are such as to justify it the hospital and the medical man are prepared to take a certain amount of risk so that he can receive an undertaking as to the limit. One of the advantages attaching to this arrangement is that there is an opportunity of impressing upon the patient the value of the services rendered to him by the medical man. In many instances, while there is complete readiness to pay any charges required by the hospital for treatment therein, there is a curious reluctance to recognise that trained and experienced skill is entitled to financial recompense.

### HEIGHTS AND WEIGHTS.

Dr. Milligan, Medical Officer of Health for Reading, gives in his annual report a comparison between the heights and weights of Reading children examined during the past three years and those of American children of similar ages. In every case, the doctor states, the advantage in both height and weight rests with the American children, a superiority which becomes more emphasised in the older groups. The average height of Reading boys of twelve years three months is given as 54¾ ins. and the weight 73 lb., as against 56¾ ins. and 79 lb. respectively in the case of American children of corresponding age. It would appear, Dr. Milligan adds, that those circumstances which assist growth in children are more favourable in America than in this country.

### MORE CANCER AMONG SPINNERS.

In a report presented at the annual meeting of the Approved Society of the Operative Spinners' Amalgamation in Manchester recently, Mr. A. Cook said that there was a great prevalence of scrotal cancer among spinners. Seven new cases had already been reported this year, which convinced some of them more than ever that this particular disease was due to the occupation.

### A LUMINOUS MAN.

The Paris correspondent of the *Daily Telegraph* writes that remarkable results have been obtained in experiments now being conducted at the International Metapsychic Institute with the Italian medium Erto, known as the "luminous man." Dr. Gustave Geley, who is being assisted in his researches by other scientists of repute, has proved that light and other emanations resembling electricity given off by Erto during his trances cannot be reproduced by any apparatus known to science. The radiations, although different from any others yet known, appear by their physical properties to be allied to X-rays and those given off by radio-active properties. A series of fifteen photographic plates have been exposed to the action of Erto's powers. On one of these, in addition to two patches of light resembling the radium light, was the imprint of a human hand. This was submitted for examination to police experts, who found that the hand-print was not that of any of the scientists present at the seance nor of the medium. The light from the medium also has the property, like X-rays, of penetrating solid obstacles, so that some plates have received its impression even when they were kept covered. The nature and origin of Erto's strange psychic manifestations constitute a problem that has so far baffled the investigators.

### DISEASE AND HIDDEN MEMORIES.

In an address before the Hampstead Council of Social Welfare, Dr. Helen Boyle, founder of the Lady Chichester Hospital, Brighton, said that many ailments and diseases were due to hidden memories or unsuspected mental troubles. In the Lady Chichester Hospital she had treated a little girl who screamed all day and refused to eat. It appeared that on the arrival of a new baby screaming and refusing food were the only means of attracting the mother's attention to herself. The cure had been effected by arousing the child's keen interest in domestic animals on a farm, and so making the child independent of the mother's attention. Children were much cleverer than adults in readjusting their environment; instinctively they resorted to cunning devices, continually making experiments, and profiting by experience. A girl was admitted to the hospital who was suffering from functional paralysis, and had not walked for two years. An only daughter, never allowed to do anything for herself, and with no personal attractions, she had accepted the part of *fainéant* and thereby attracted the attention she coveted. In hospital she found sympathy, was told she could walk, and in a week was walking normally.

